# Genetic analysis of 1051 Chinese families with Duchenne/Becker Muscular Dystrophy

**DOI:** 10.1186/s12881-019-0873-0

**Published:** 2019-08-14

**Authors:** Xiangdong Kong, Xingjian Zhong, Lina Liu, Siying Cui, Yuxia Yang, Lingrong Kong

**Affiliations:** The Genetics and Prenatal Diagnosis Center of the First Affiliated Hospital of Zhengzhou University, No. 1, Jianshe East Rd, Erqi District, Zhengzhou, Henan Province China

**Keywords:** Duchenne muscular dystrophy (*DMD*), Gene mutations, Multiplex ligation-dependent probe amplification (MLPA), Next-generation sequencing (NGS), Sanger sequencing

## Abstract

**Background:**

Duchenne Muscular Dystrophy (DMD) is the most common muscle disease in children, and there are no effective therapies for DMD or Becker Muscular Dystrophy (BMD). Currently, targeted gene therapy treatments have emerged. As a result, genetic diagnosis is the basis of treatment. In addition, genetic and prenatal diagnosis significantly reduces their incidence rates. This study combines the application of multiplex ligation-dependent probe amplification technology (MLPA) and “next-generation” sequencing technology (NGS) as the most economical and efficient method of diagnosis. Therefore, in the diagnosis of DMD/BMD, patients’ MLPA data are first used to detect *DMD* gene deletions or duplications, and NGS and Sanger sequencing are then applied to exclude MLPA-negative samples. Meanwhile, polymerase chain reaction (PCR) is used to detect single exon deletions to exclude false-positives in MLPA caused by point mutations.

**Methods:**

In this study, we recruited 1051 proband families of DMD from 2016 to 2018 and had access to information that could identify individual participants during or after data collection. Patients who were diagnosed with DMD were first tested by MLPA. MLPA results with single exon deletions were validated with PCR amplification and Sanger sequencing. The negative results of MLPA were further analysed with NGS and validated by Sanger sequencing. For novel missense mutations, phenotype-genotype correlations were analysed using PolyPhen2 and mutation taster. All methods were performed in accordance with the relevant guidelines and regulations.

**Results:**

*DMD* mutations were identified in 1029 families (97.91%, 1029/1051). Large deletions, duplications, and small mutations accounted for 70.41% (740/1051), 8.28% (87/1051), and 19.12% (201/1051) of all cases, respectively. There were 205 small mutation types, 53 of which were novel. The rate of de novo mutations was 39.45% (187/474) and was higher in large duplications (49.53%, 157/317). Among 68 asymptomatic patients (< 3 years old) with unexplained persistent hyperCKaemia upon conventional physical examination, 63 were diagnosed as DMD/BMD according to genetic diagnosis.

**Conclusion:**

Our results expand the spectrum of *DMD* mutations, which could contribute to the treatment of DMD/BMD and provide an effective diagnosis method. Thus, the combination of MLPA, NGS and Sanger sequencing is of great significance for family analysis, gene diagnosis and gene therapy.

## Background

Duchenne muscular dystrophy (DMD, OMIM: 310200), the most common X-linked recessive inherited muscle disease, affects approximately 1 in 3600–6000 live male births [1–3]. The age of diagnosis is approximately 5 years when early symptoms occur [4]. Affected children rely on wheelchairs approximately 12 years of age with progressive muscle weakness, and they often die of respiratory or cardiac failure around the second decade of life. Compared with DMD, BMD (OMIM: 300376) is milder, with later symptom occurrence, slower disease progression, and fewer effects on survival; however, it results in decreased quality of life. DMD is caused by structural and functional changes of dystrophin induced by mutations of the *DMD* gene (OMIM: 300377), which is located on Xp21.1 and represents the largest known gene in humans. The *DMD* gene spans approximately 2.4 Mb of genomic DNA and contains 79 exons and 78 introns, generating a 14 kb mRNA transcript, which may explain its high mutation rate [5]. Approximately 60–70% of DMD/BMD cases are caused by deletions or duplications of one or more exons in the *DMD* gene. In this study, we analysed the genetic mutations of 1051 unrelated Chinese DMD/BMD families, clarified the distribution characteristics of *DMD* gene mutations in the Chinese Han population, and explored the detection strategy of *DMD* gene mutations. In clinical practice, MLPA has been widely used to detect such mutations. The remaining 25–35% of small mutations, including missense, splice site, nonsense, and frameshift mutations, require Sanger sequencing or NGS for diagnosis. Because of its high throughput and low cost, which compensates for the deficiency of Sanger sequencing, NGS has prominent advantages in detecting *DMD* gene mutations [6].

## Methods

### Subjects

In total, 1051 unrelated DMD/BMD families were recruited from January 1st, 2016 to November 31st, 2018 in the Genetic and Prenatal Diagnosis Center of the First Affiliated Hospital of Zhengzhou University. The inclusion criteria for DMD patients (probands) in this study were: [1] clinical manifestations of progressive muscle weakness, motor function regression and positive Gower’s sign; [2] history of gastrocnemius pseudo-hypertrophy; [3] abnormal increase in serum creatine kinase; [4] electromyogram (EMG) showing myogenic damage or muscle biopsy revealing a change in dystrophin-deficient muscular dystrophy; [5] other inherited neuromuscular diseases, such as myasthenia gravis and spinal muscular atrophy, were excluded. The first step of genetic diagnosis is MLPA, which can determine if the DMD gene is deleted or duplicated. Negative MLPA results in the case of the above clinical symptoms were further analysed with NGS and validated by Sanger sequencing. For novel missense mutations, phenotype-genotype correlations were analysed using PolyPhen2 and MutationTaster. The clinical manifestations of BMD patients were similar to those of DMD patients, but with later onset time, slower disease progression, and milder symptoms. DMD is a serious X-linked, recessive, inherited fatal disease. Thus, the incidence rate of women is much lower than men. That is why there are far more male patients than female patients in our data. Most male patients cannot survive to marriage. Therefore, once a patient is diagnosed, only their mother’s *DMD* gene must generally be checked. All patients and their family members signed informed consent. This study was approved by the Ethics Committee of the First Affiliated Hospital of Zhengzhou University.

### DNA sample preparation

Peripheral blood samples (2~5 ml per individual) were extracted from probands or their mothers in families with a deceased proband or sporadic patient, using ethylene diamine tetraacetic acid (EDTA) as an anti-coagulant. Genomic DNA was isolated with a commercial kit (TIANamp DNA Kit, Tiangen Biotech, Beijing, China). The Qubit dsDNA HS Assay kit (Life Technologies Co., LTD) was used to assess DNA.

## Methods

### MLPA analysis

MLPA was performed with a SALSA MLPA Kit P034/P035 *DMD*/Becker (MRC-Holland, Amsterdam, The Netherlands) according to the manufacturer’s protocol. Amplification products were analysed by capillary electrophoresis on an ABI 3130 genetic analyser. The original data were analysed by Genemapper 4.0 and Coffalyser.net software, and the copy number was calculated according to the MLPA kit instructions.

### Next-generation sequencing (NGS) and data analysis

A custom *DMD* panel was designed using Ion AmpliSeq™ designer (www.ampliseq.com) to ensure all 79 exons and exon/intron junction regions (10 bp) were directly sequenced. Amplification was carried out with the Ion AmpliSeq™ Library kit 2.0, and template preparation was performed by the Ion PGM™ Template OT2 200 kit and Ion OneTouch instrument. Template enrichment and separation was based on the Dynabeads MyOne Streptavidin C1 bead kit and Ion OneTouch ES instrument, and sequencing-by-synthesis reactions were carried out with the Ion PGM™ Sequencing 200 kit v2 on an Ion PGM sequencing platform (the sequencing reaction was 500 flows). After sequencing, the Ion Torrent Suite 4.0.2 software was used for data processing. VCF files were downloaded and variation annotations performed with Ion reporter software (https://ionreporter.lifetechnologies.com/ir/). Base mutations, including *DMD* gene indels and substitutions, were screened and compared in the NCBI dbSNP-, Hapmap- and 1000 genome-databases for the exclusion of known polymorphic loci. For missense mutations, PROVEAN, PolyPhen2 and MutationTaster software were used to predict protein functions, and conservative analysis of amino acids in human, macaque, dog, mouse, toad, and other species was carried out using the UCSC database. The *DMD* mutations database of Leiden Open Variation (LOVD: http://www.lovd.nl/) was used to determine whether the mutations had been reported.

### PCR amplification and sanger sequencing

Sanger sequencing was performed on an ABI 3130XL DNA Analyzer (Thermo Fisher Scientific, USA), and the results were analysed using ABI sequencing Analysis 5.1.1 and mapped with the standard *DMD* reference sequence (GenBank transcript ID: NM_004006). People with suspected and established pathogenic mutations of NGS or single exon deletions of MLPA and 100 controls were sequenced to confirm the identified mutations.

## Results

### Types of DMD gene mutations

In total, 1051 Chinese DMD/BMD families, including 1022 male and 29 female probands, were assessed in this study. Among the 1051 cases, *DMD* gene mutations were identified in 1029 cases, with a detection rate of 97.91% (1029/1051). Large deletions and duplications accounted for 70.41% (740/1051) and 8.28% (87/1051), respectively. Among the remaining 201 patients, 205 small mutations were revealed with DNA sequencing (Table 1).

**Table 1:** Frequency of different *DMD* mutations in 1051 families with DMD/BMD

Type of mutation	Number of families with DMD(n = 1003)	Number of families with BMD(n = 26)	Total(n = 1051)
Deletion	721 (71.88%)	19 (73.08%)	740 (70.41%)
Duplication	86 (8.57%)	1 (3.85%)	87 (8.28%)
Deletion and duplication	0 (0%)	1 (3.85%)	1 (0.10%)
Small mutation	196 (19.54%)	5 (19.23%)	201 (19.12%)

### Families with large deletions in the DMD gene

The most common exon deletion pattern was the deletion of exons 45–50 (45/740, 6.08%). A hotspot region of large exon deletions in patients with DMD was observed between exons 44 and 52 (479/740, 64.73%).

#### Families with large duplications in the DMD gene

Single exon 2 duplications (9/87, 10.34%) were the most frequent pattern. The largest duplications in our study were observed in the hotspot region between exons 2 and 20 (36/87, 41.38%).

#### Small *mutations of the DMD gene*

The spectrum of identified small mutations is shown in Table 2, including 102 (49.76%, 102/205) nonsense mutations, 58 (28.29%, 58/205) small insertions or deletions, 30 (14.63%, 30/205) splice-site mutations, and 15 (7.32%, 15/205) missense mutations. Most of them were predictable termination codons or splice defects. Although some mutation types were detected two or three times in different families, no mutation hotspots were observed. Among the 205 mutations, 53 mutations had not previously been reported (Table 3).

**Table 2:** Frequency of small mutations at DMD

Type of small mutation	Frequency in families with DMD(n = 200)	Frequency in families with BMD(n = 5)	Total (n = 205)*
Missense	13	2	15 (7.32%, 15/205)
Frameshift	57	1	58 (28.29%, 58/205)
Nonsense	100	2	102 (49.76%, 102/205)
Splice sites	30	0	30 (14.63%, 30/205)

*:The detection frequency of small mutations was 205 in 201 families. Among them, more than one small mutation types was found in three families respectively. One families had splicing mutations c.94-9dupT and nonsense mutation c.100A > T(p.Lys34*), one families had missense mutations c.8729A > T(p.Glu2910Val) and c.8734A > G(p.Asn2912Asp) and the other families had splice sites mutation c.9164-2A > G and missense mutations c.5163G > C, c.3226C > G

**Table 3:** Novel mutations

No.	Types	Location	Mutation	Protein
1	Missense	E24	c.3226C > G	p.Pro1076Ala
2	Frameshift	E3	c.96delT	p.Phe32Leufs*19
3	Frameshift	E4	c.193_194delGA	p.Glu65fs*23
4	Frameshift	E6	c.360_361insC	p.Lys121fs*
5	Frameshift	E11	c.1152delG	p.Gly384fs*3
6	Frameshift	E11	c.1198_1199insA	p.Leu400Hisfs*6
7	Frameshift	E11	c.1206_1207delGGinsAT	p.Gly403*
8	Frameshift	E11	c.1327_1328insA	p.Ser443Lysfs5*
9	Frameshift	E20	c.2496delinsTT	p.Ile833Tryfs*3
10	Frameshift	E23	c.3075_3075delT	p.Ile1025Metfs*19
11	Frameshift	E26	c.3569delC	p.Pro1190Glnfs11*
12	Frameshift	E27	c.3728_3729insT	p.Leu1243Leufs*11
13	Frameshift	E29	c.3988delC	p.Leu1330fs*10
14	Frameshift	E33	c.4583delA	p.Gln1528fs*18
15	Frameshift	E36	c.5100_5101delAC	p.Leu1701Phefs*2
16	Frameshift	E43	c.6128_6131delATAG	p.Asp2043fs*29
17	Frameshift	E45	c.6472_6473delGT	p.Val2158fs*
18	Frameshift	E47	c.6791delA	p.Gln2264Argfs*7
19	Frameshift	E51	c.7431_7434delGGCTinsCA	p.Arg2477fs*13
20	Frameshift	E55	c.8215_8216insT	p.Gln2739Serfs*6
21	Frameshift	E59	c.8681_8682delA	p.Glu2895fs*14
22	Frameshift	E64	c.9358delT	p.Cys3120fs*
23	Frameshift	E21	c.2673_2674delAA	p.Lys891fs*9
24	Nonsense	E7	c.620C > T	p.Leu207*
25	Nonsense	E12	c.1375G > T	p.Glu459*
26	Nonsense	E12	c.1396A > T	p.Lys466*
27	Nonsense	E13	c.1510C > T	p.Gln504*
28	Nonsense	E15	c.1729G > T	p.Gln577*
29	Nonsense	E20	c.2556G > A	p.Trp852*
30	Nonsense	E23	c.3106G > T	p.Gln1036*
31	Nonsense	E24	c.3172C > T	p.Gln1058*
32	Nonsense	E25	c.3346A > T	p.Lys1116*
33	Nonsense	E27	c.3655G > T	P.Gln1219*
34	Nonsense	E29	c.3923C > A	p.Ser1308*
35	Nonsense	E33	c.4656 T > A	p.Tyr1552*
36	Nonsense	E34	c.4729C > T	p.Arg1577*
37	Nonsense	E42	c.6025C > T	p.Gln2009*
38	Nonsense	E45	c.6550A > T	p.Lys2184*
39	Nonsense	E46	c.6739A > T	p.Lys224*
40	Nonsense	E48	c.7029G > A	p.Trp2343*
41	Nonsense	E51	c.7455G > A	p.Trp2485*
42	Nonsense	E55	c.8197G > T	p.Glu2733*
43	Nonsense	E63	c.9277C > T	p.Gln3093*
44	Splice sites	E3	c.186 + 2 T > G	–
45	Splice sites	E11	c.1150-2A > G	–
46	Splice sites	E20	c.2622 + 1_2622 + 5delGTAAG	–
47	Splice sites	E28	c.3921 + 12A > G	–
48	Splice sites	E41	c.5922 + 4A > T	–
49	Splice sites	E43	c.6290 + 5G > T	–
50	Splice sites	E55	c.8027-2A > G	–
51	Splice sites	E55	c.8217 + 2 T > C	–
52	Splice sites	E62	c.9164-2A > G	–
53	Splice sites	E65	c.9362-2A > C	–

The novel missense mutation c.3226C > G was detected with two other mutations (c.5163G > C and c.9164-2A > G) in one proband. The mutation type c.5163G > C had been recorded as benign. The mutation c.3226C > G was predicted to be damaging, with a score of 0.989 in HumDiv of PolyPhen2, and predicted to be disease causing with MutationTaster.

### ***C***arrier screening

In this study, the mothers of living probands in 474 families were screened, 287 of which carried the same *DMD* gene mutations as the respective probands, indicating an inherited mutation rate of 60.55% (287/474). The remaining 187 mothers did not carry the same mutations with probands, with a de novo mutation rate of 39.45% (187/474). Among these 187 cases, the de novo mutation rate of large *DMD* gene deletions, large duplications and small mutations was 49.53% (157/317), 18.75% (6/32) and 19.2% (24/125), respectively (Table 4). Interestingly, among these 187 families, the same mutations were detected in 7 of the probands’ brothers or sisters. Germline mosaicism could be the most possible reason, which indicated that the incidence rate of germline mosaicism was 1.48% (7/474).

**Table 4:** Genetic characteristics analysis of DMD gene mutations

Types of mutations	de novo mutations	Inherited pathogenic variants	Rate of de novo mutations
Large deletions	157	160	49.53%
Large duplications	6	26	18.75%
Small mutations	24	101	19.2%
Total	187	287	39.45%

## Discussion

The diagnosis of *DMD* gene mutations can support its treatment. In this study, we analysed 1051 Chinese families with DMD/BMD; 1029 (97.91%) patients were identified to have genetic mutations, which should be considered as the largest *DMD* gene mutation report in China. Among these families, 740 (70.41%) probands were large deletions, which occupied the most mutation proportions, and 87 (8.28%) were duplications, which corroborated the results of the Leiden database (http://www.dmd.nl/) (large deletions, 72%; large duplications, 8%) and TREAT-NMD *DMD* Global Database [7] (large deletions, 68%; large duplications, 11%). According to the *DMD* genomic structure, it is possible to treat DMD by restoring the ORF of an out-of frame deletion by splicing out the exon. Eteplirsen, developed by Sarepta to skip exon 51, was recently granted accelerated approval by the Food and Drug Administration (FDA). Based on our results of exon deletions, we can easily obtain a clear message about its applicability (Table 4).

De novo mutations cannot be ignored when performing genetic counselling. In this study, a de novo mutation rate of 39.45% (187/474) was obtained. The de novo mutation rate for large deletions (49.53%, 157/317) was highest compared with other mutations, corroborating a study on prenatal diagnosis in 131 Chinese families with *DMD/BMD* in 2017 [8] (51.1% of probands with large *DMD* gene deletions had de novo mutations). The mechanism of de novo mutations is not yet fully understood, but germline mosaicism could be one possible reason. Therefore, an effective and expeditious diagnosis method and a systematic pedigree analysis are necessary for genetic counselling of DMD.

MLPA is one of the most widely used methods. It can accurately and rapidly detect large deletion and duplication mutations of the *DMD* gene. The main limitation of MLPA is its inability to detect non-deletion and non-duplication mutations. Gene deletion or duplication is analysed by MLPA based on probe amplification, but the probe cannot be combined with DNA with small mutations, resulting in loss of amplification and yielding false-positive results. NGS can detect all mutation types and has the advantages of high throughput, short time and abundant data. However, the cost is higher when facing exon deletions/duplications of the *DMD* gene. Therefore, the combination of MLPA and NGS is the most economical and efficient method for diagnosis. In our study, the patient’s MLPA data were first used to detect *DMD* gene deletions or duplications, and NGS and Sanger sequencing were then applied to exclude MLPA-negative samples. Meanwhile, PCR was applied for detection of single exon deletions to exclude false-positives in MLPA caused by point mutations.

DMD is a serious X-linked, recessive, inherited, fatal disease but often shows mild symptoms prior to the age of 5. Therefore, the diagnosis of female *DMD* mutation carriers and children is considered very important. The majority of female *DMD* mutation carriers have no significant clinical signs. Symptomatic female *DMD* carriers show symptoms in childhood. In recent years, multiple studies have explored the possible pathogenetic mechanisms of symptomatic DMD in female carriers, including skewed X-inactivation [9], X/autosomal translocation [10], germline mosaicism [11], uniparental disomy in the X chromosome [12], and Turner syndrome with *DMD* [13], with the daughter having female skewed X chromosome inactivation. Overall, 29 DMD female patients were involved in this study, but no heredity predisposition to skewed X chromosome inactivation was found. Therefore, we believe that skewed X chromosome inactivation is likely to occur randomly. In addition, 92.65% (63/68) of asymptomatic patients (< 3 years old) with unexplained persistent hyperCKaemia enrolled in this study were diagnosed with *DMD* mutations. Therefore, serum CK screening for newborns is an effective screening method to identify suspicious patients.

*DMD* is one of the largest human genes and has several mutation types, including large fragment deletions or duplications (≥1 exon) and small mutations. Therefore, it is difficult to unify the clinical diagnosis methods of DMD/BMD patients. At present, the methods of genetic testing for the *DMD* gene include PCR amplification, multiplex PCR, Sanger sequencing, real-time PCR, MAPH, MLPA, and NGS [14, 15].

As for small mutations, there were no differences between our results and those of Mariko Okubo et al. [16] in Japan, who demonstrated that there are no racial differences between *DMD* mutations. Unlike the hotspot of exon deletions/duplications, there were no mutation hotspots, which means individualized treatment strategies are needed.

In this study, mutations could not be detected in 22 families, which may be due to rearrangements in introns or the 3’or 5′ untranslated regions (UTRs). Further consideration should be given to whole genome sequencing and muscular biopsy, and possible clinical symptoms caused by other neuromuscular diseases cannot be ruled out.

## Conclusion

This dataset suggests that the combination of MLPA, NGS and Sanger sequencing is an efficient gene diagnostic tool for DMD/BMD and provides a useful reference to further the diagnosis and treatment of DMD.

## Data Availability

The datasets generated and/or analysed during the current study are not publicly available due the file is including a larger number of high-throughput sequencing that is too large to upload but are available from the corresponding author on reasonable request.

## Abbreviations

BMD: Becker Muscular Dystrophy
DMD: Duchenne Muscular Dystrophy
MLPA: Multiplex Ligation-dependent Probe Amplification Technology
NGS: “Next-generation” Sequencing
